# Intracervical insemination versus intrauterine insemination with cryopreserved donor sperm in the natural cycle: a randomized controlled trial

**DOI:** 10.1093/humrep/deac071

**Published:** 2022-04-23

**Authors:** P A L Kop, M van Wely, A Nap, A T Soufan, A A de Melker, B W J Mol, R E Bernardus, M De Brucker, P M W Janssens, J J P M Pieters, S Repping, F van der Veen, M H Mochtar

**Affiliations:** 1 Department of Obstetrics and Gynaecology, Center for Reproductive Medicine, Amsterdam Reproduction & Development Institute, Amsterdam UMC, University of Amsterdam, Amsterdam, The Netherlands; 2 Department of Obstetrics and Gynaecology, Rijnstate, Arnhem, The Netherlands; 3 Department of Obstetrics and Gynaecology, Monash University, Clayton, Australia; 4 Aberdeen Centre for Women’s Health Research, Institute of Applied Health Sciences, School of Medicine, Medical Sciences and Nutrition, University of Aberdeen, Aberdeen, UK; 5 Nij Barrahûs, Wolvega, The Netherlands; 6 University Hospital Brussels, Brussels, Belgium; 7 Fertility Clinic, Vivaneo Medisch Centrum Kinderwens Leiderdorp, Leiderdorp, The Netherlands

**Keywords:** IUI, intrauterine insemination, intracervical insemination, donor sperm, donor sperm treatment, natural cycle, cryopreserved donor sperm, live birth rate

## Abstract

**STUDY QUESTION:**

Is intracervical insemination (ICI) non-inferior to IUI with cryopreserved donor sperm in the natural cycle in terms of live birth?

**SUMMARY ANSWER:**

ICI with cryopreserved donor sperm in the natural cycle was inferior to IUI in terms of live birth.

**WHAT IS KNOWN ALREADY:**

Both ICI and IUI in the natural cycle are performed as first-line treatments in women who are eligible for donor sperm treatment. High-quality data on the effectiveness of ICI versus IUI with cryopreserved donor sperm in the natural cycle in terms of live birth is lacking.

**STUDY DESIGN, SIZE, DURATION:**

We performed an open-label multicentre randomized non-inferiority trial in the Netherlands and Belgium.

**PARTICIPANTS/MATERIALS, SETTING, METHODS:**

We randomly allocated women who were eligible for donor sperm treatment with cryopreserved donor semen to six cycles of ICI in the natural cycle or six cycles of IUI in the natural cycle. The primary outcome was conception within 8 months after randomization leading to a live birth. Secondary outcomes were ongoing pregnancy, multiple pregnancy, clinical pregnancy, miscarriage and time to conception leading to live birth. We calculated relative risks (RRs) and risk differences (RDs) with 95% CI. Non-inferiority would be shown if the lower limit of the 95% RD CI was <−12%.

**MAIN RESULTS AND THE ROLE OF CHANCE:**

Between June 2014 and February 2019, we included 421 women, of whom 211 women were randomly allocated to ICI and 210 to IUI. Of the 211 women allocated to ICI, 2 women were excluded, 126 women completed treatment according to protocol and 75 women did not complete 6 treatment cycles. Of the 210 women allocated to IUI, 3 women were excluded, 140 women completed treatment according to protocol and 62 women did not complete 6 treatment cycles. Mean female age was 34 years (SD ±4) in both interventions. Conception leading to live birth occurred in 51 women (24%) allocated to ICI and in 81 women (39%) allocated to IUI (RR 0.63, 95% CI: 0.47 to 0.84). This corresponds to an absolute RD of −15%; 95% CI: −24% to −6.9%, suggesting inferiority of ICI. ICI also resulted in a lower live birth rate over time (hazard ratio 0.58, 95% CI: 0.41–0.82). Our per-protocol analysis showed that, within the 8 months treatment horizon, 48 women (38%) had live births after ICI and 79 women (56%) had live births after IUI (RR 0.68, 95% CI: 0.52–0.88; RD −18%, 95% CI: −30% to −6%).

**LIMITATIONS, REASONS FOR CAUTION:**

The study was non-blinded owing to the nature of the interventions. We consider it unlikely that this has introduced performance bias, since pregnancy outcomes are objective outcome measures.

**WIDER IMPLICATIONS OF THE FINDINGS:**

Since ICI in the natural cycle was inferior to IUI in the natural cycle with cryopreserved donor sperm in terms of live birth rate, IUI is the preferred treatment.

**STUDY FUNDING/COMPETING INTEREST(S):**

This trial received funding from the Dutch Organization for Health Research and Development (ZonMw project number 837002407). B.W.J.M. is supported by an NHMRC Investigator grant (GNT1176437), reports consultancy for ObsEva and has received research funding from Guerbet, Ferring and Merck. The other authors do not declare a COI.

**TRIAL REGISTRATION NUMBER:**

NTR4462

**TRIAL REGISTRATION DATE:**

11 March 2014

**DATE OF FIRST PATIENT’S ENROLMENT:**

03 June 2014

## Introduction

Inseminations with cryopreserved donor sperm are widely performed. In Europe, approximately 49 000 cycles were reported by ESHRE in 2015 (De Geyter *et al.*, 2020). The National Perinatal Epidemiology and Statistics Unit reported 3262 cycles with donor sperm in Australia and New Zealand in 2018 (Newman *et al.*, 2020). Data on other continents are lacking.

To prevent transmission of sexually transmitted diseases, the use of cryopreserved donor semen which is quarantined until the donor is tested negative for HIV, hepatitis B, C and other venereal diseases, is mandatory (EuropeanUnion, 2004; ASRM, 2013; NICE, 2013). The downside of cryopreserved semen is that pregnancy rates are lower compared to inseminations with fresh semen owing to an adverse effect of freezing and thawing on sperm motility (Leeton *et al.*, 1980; Richter *et al.*, 1984; Keel *et al.*, 1987).

Donor sperm treatment can be carried out by intracervical insemination (ICI) or by IUI. The only guideline on the practice of donor sperm treatment, published by the National Institute for Health and Care Excellence (NICE), recommends IUI in the natural cycle for six cycles (NICE, 2013). The evidence upon which this guideline is based is weak and of low quality. Only two small randomized controlled trials (RCTs) of low quality compared ICI and IUI in the natural cycle, with only one trial reporting live birth as an outcome in just 26 women (Patton *et al.*, 1992; Hurd *et al.*, 1993).

In our Cochrane review, we subsequently pooled the data of these two studies and reported that there is insufficient evidence of a difference in live birth between ICI and IUI in the natural cycle (one study, 26 women, odds ratio (OR) 3.24, 95% CI: 0.12 to 87.13), although IUI might result in higher clinical pregnancy rates compared to ICI (two studies, 76 women, OR 6.18, 95% CI: 1.91 to 20.03) (Kop *et al.*, 2018). The only available large retrospective cohort study suggests similar cumulative ongoing pregnancy rates after six cycles of ICI and IUI in the natural cycle (1843 women, hazard ratio (HR) 1.02 95% CI: 0.84 to 1.23). Live birth rate was not reported (Kop *et al.*, 2015).

In view of this lack of evidence, we aimed to study the effectiveness of ICI compared to IUI in the natural cycle in women who started inseminations with cryopreserved donor sperm.

## Materials and methods

### Study design

This study was an open-label multicentre, randomized controlled non-inferiority trial among five fertility clinics in the Netherlands and one in Belgium. We recruited women between June 2014 and February 2019. The Medical Ethical Committee of the Academic Medical Centre and the Dutch Central Committee on Research involving Human Subjects approved this study (CCMO NL 47330-018-13) and the board of directors of each participating site approved local execution. The trial was registered at the Dutch trial register (NTR 4462). The protocol was published previously (Kop *et al.*, 2019).

### Study population

All women with an indication for donor sperm treatment were eligible for the study. Indications for donor sperm treatment were obstructive and non-obstructive azoospermia in heterosexual couples, severely impaired semen quality in couples who did not wish to undergo or had not been successful with ICSI, prevention of vertical transmission of a genetic defect or prevention of transmission of HIV in couples who did not wish to try natural conception or semen washing. In addition, lesbian couples or single women who applied for donor sperm treatment were eligible to participate.

Women had to be between 18 and 43 years of age with a regular menstrual cycle, or ovulatory after ovulation induction in women with normogonadotrophic anovulation. Women with normogonadotrophic anovulation started ovulation induction according to local protocol with clomiphene citrate or letrozole. After ovulation was detected by a basal temperature chart or ultrasound monitoring, women could start insemination with donor sperm in the next cycle.

Women with known double-sided tubal pathology, irregular menstrual cycles, or IVF or IUI in their history were not eligible. Heterosexual couples after failed ICSI or ICSI with testicular sperm extraction were excluded from the study if there was a history of female factors, such as low ovarian response during ICSI treatment.

### Sperm donors

In the Netherlands, all sperm donors were non-anonymous according to national legislation (Staatsblad, 2002). In Belgium, sperm donors could be non-anonymous or anonymous (Senaat, 2005-2006). The screening and selection procedure was performed according to local protocols, adapted from the European Union tissue directive (EU 2004/23/EC).

### Interventions

We treated couples for a maximum of six cycles within a time horizon of 8 months. Ovulation was detected by urinary or serum LH tests or transvaginal sonography, depending on the local protocol of the fertility clinic. In case of monitoring with urinary LH tests, women tested their urine once per day, starting on an individually calculated cycle day based on their basal body temperature chart of the previous cycle. Insemination followed within 24 h of LH detection in urine. In case of monitoring with serum LH measurements, women were tested from cycle Day 11 onwards and insemination followed within 24 h of the serum LH rise. In case of monitoring by transvaginal sonography, women were followed until a dominant follicle was present with a diameter of at least 16 mm. Insemination followed after ovulation triggering by hCG (Pregnyl, Organon, Oss, the Netherlands) 36–40 h thereafter.

In the ICI cycles, inseminations were performed with unprocessed cryopreserved semen by cap or by straw. In the IUI cycles, inseminations were performed with processed semen. The processing could be done in one of two ways depending on the local laboratory protocol. The first technique was that the unprocessed semen was first cryopreserved and thawed, and then processed against a density gradient centrifugation and/or a washing step with culture medium. The second technique was that semen was first processed against a density gradient centrifugation and/or a washing step with culture medium and then cryopreserved.

All inseminations were performed in the clinics.

Women were treated for a maximum of six cycles or until pregnancy occurred within a time horizon of 8 months. Clinical and ongoing pregnancies were confirmed by ultrasound.

### Outcome measures

The primary outcome was conception leading to live birth per woman, defined as any baby born alive with a gestational age beyond 24 weeks. Pregnancies that occurred within the first 8 months after randomization counted for assessment of the primary outcome.

Secondary outcomes were clinical pregnancy defined as any registered heartbeat on ultrasound, ongoing pregnancy defined as a positive heartbeat at or beyond 12 weeks of gestation, miscarriage defined as registered heartbeat before 12 weeks of gestation, multiple pregnancy defined as registered heartbeat of at least two foetuses at 12 weeks of gestation, ectopic pregnancy, congenital anomalies defined as structural or functional anomalies that occur intrauterine and can be identified prenatally or at birth, and time from randomization leading to the birth of a live child.

### Sample size calculation

We designed the study as a non-inferiority trial. We assumed live birth rates of 40% after six cycles ICI and IUI, based on our retrospective cohort (Kop *et al.*, 2015). To exclude a non-inferiority margin of 12% to the detriment of ICI—by a one-sided *Z* test (unpooled) with an 80% power and 5% alpha-, we needed to recruit 208 women per treatment.

### Randomization and masking

Eligible women were informed about the study by their doctor or by a research nurse. After providing written informed consent, women were randomized using a central password protected Internet-based randomization program.

The randomization list had been prepared by an independent statistician with a variable block size with randomly selected block sizes that varied between two, four and six. There was no stratification. Neither the recruiters nor the trial project group could access the randomization sequence.

### Statistical analysis

We analysed all outcomes on an intention-to-treat (ITT) basis. For live birth, we tested non-inferiority on the basis of the absolute risk difference (RD) with the absolute left boundary margin of 12%. We also expressed differences as absolute RDs and risk rate with 95% CI and used Chi-square test for formal analysis, and did the same for secondary endpoints. To account for time to conception leading to live birth, we constructed cumulative hazard curves using the log-rank test to compare the interventions and calculated the corresponding hazard rates with 95% CI. We constructed a hazard curve showing time to conception leading to live birth over six cycles and a hazard curve showing time to conception leading to live birth over 8 months. For the primary outcome, we also performed a per-protocol analysis, which was not pre-planned. The per-protocol analysis was limited to women who were treated according to the assigned intervention, who did not switch treatment and who had completed six inseminations in case of treatment failure. We performed an analysis on pre-specified subgroups possibly affecting our primary outcome. Variables considered in the analysis were women aged under or over 35 years of age and women being nulliparous or multiparous. Results were expressed as relative risks (RRs) with corresponding 95% CIs. Analyses were performed using IBM SPSS Statistics for windows version 26 (Armonk, NY, USA: IBM Corp).

### Data safety monitoring board

A pre-planned independent interim analysis was performed by the Data Safety Monitoring Board of the Dutch Consortium for Women’s Health Research when 200 women were included to exclude large differences in conception leading to live birth (Haybittle–Peto with *P* ≤ 0.001). The Data Safety Monitoring Board advised to proceed with the trial as planned.

## Results

Between June 2014 and February 2019, we recruited 421 women of whom 211 women were allocated to ICI and 210 women to IUI. We excluded five women after randomization but before starting treatment; three women because they did not fulfil the inclusion criteria and two women because they withdrew their informed consent.

**Figure 1.**
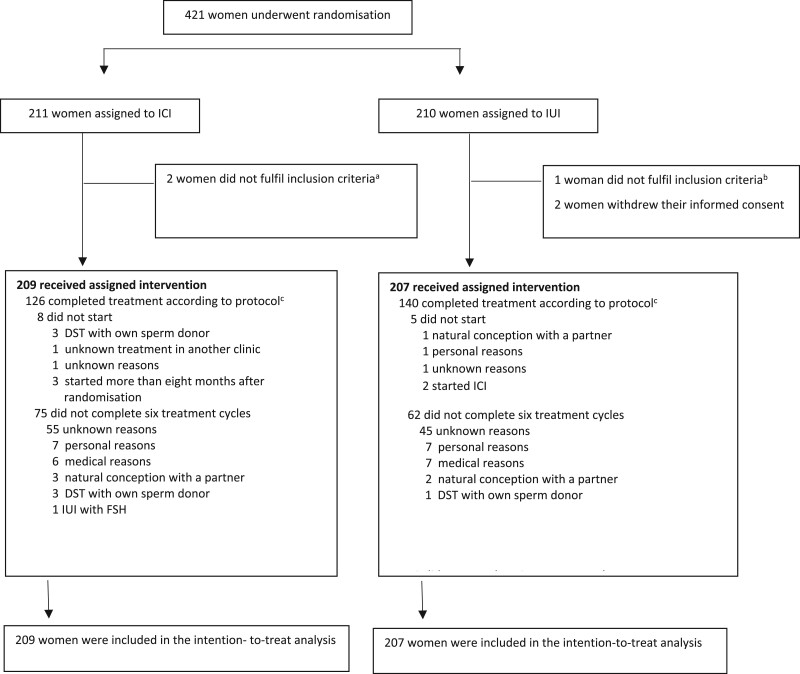
**Profile of the randomized controlled trial comparing ICI with IUI using cryopreserved donor sperm in a natural cycle.** ^a^One woman had bilateral tubal pathology, one woman was randomized a second time after a live birth after the assigned intervention. ^b^One woman was randomized a second time after a live birth after the assigned intervention. ^c^Treatment according to protocol: women who did not switch treatment and who had completed six inseminations in case of treatment failure. ICI, intracervical insemination; DST, donor sperm treatment.

For the ITT analysis, we could include 209 women allocated to ICI and 207 women allocated to IUI (Fig. 1).

The baseline characteristics were well balanced between women, except for nulliparity that was present in 77% of women allocated to ICI versus 87% of women allocated to IUI (Table I). One hundred and ninety-six women (94%) allocated to ICI and 196 women (95%) allocated to IUI were inseminated with donor sperm of a Dutch sperm bank.

**Table I:** Baseline characteristics of participants in a randomized controlled trial comparing ICI with IUI using cryopreserved donor sperm in a natural cycle.

Characteristics	ICI (n = 209)	IUI (n = 207)
**Women**		
Mean female age (years)	34.4 ± 3.8	34.4 ± 3.9
Nulliparous	161 (77)	180 (87)
Indication for donor sperm treatment		
Heterosexual couples:	47 (23)	48 (23)
Azoospermia, did not opt for TESE	4	8
No sperm after TESE	28	24
Partner had vasectomy	0	1
No pregnancy after ICSI-TESE	8	5
No pregnancy after ICSI	2	3
Partner carrier genetic defect	4	6
Poor embryo quality after ICSI	0	1
Unknown	1	0
Lesbian couples	69 (33)	58 (28)
Single women	93 (44)	101 (49)
Current smoking status (yes)	24 (11)	34 (16)
Normogonadotropic anovulation	1 (0.5)	1 (0.5)
Mean BMI in kg/m^2^	25 (17–36)	24 (16–43)
Mean (range) total motile sperm count (×10^6^)	5.9 (1.05–24.3)	5.7 (0.52–26.7)

ICI, intracervical insemination; TESE, testicular sperm extraction.

Data are n (%), mean (SD).

In ICI, inseminations were performed with donor sperm with a median total motile sperm count of 5.9 × 10^6^ (quartiles: 1.05–24.3 × 10^6^) per sample and in IUI with donor sperm with a median total motile sperm count of 5.7 × 10^6^ (quartiles: 0.52–26.7 × 10^6^) per sample.

Pregnancy outcomes are summarized in Table II. Within the 8 months treatment horizon, there were 51 live births in 209 women (24%) after ICI and 81 live births in 207 women (39%) after IUI (RR 0.62, 95% CI: 0.47–0.84). This corresponds to an absolute RD of −15% (95% CI: −24% to −6.9%) and to a 90% left boundary of −22%, thus crossing the pre-set absolute difference of 12%.

**Table II:** Pregnancy outcomes after the 8-month study period per woman randomized.

	ICI (n = 209)	IUI (n = 207)	Relative risk (95% CI)
Live birth	51 (24)	81 (39)	0.62 (0.47–0.84)
Ongoing pregnancy	52 (25)	82 (39)	0.62 (0.46–0.82)
Clinical pregnancy	61 (29)	95 (46)	0.64 (0.49–0.82)
Multiple pregnancy	1 (0.5)	1 (0.5)	1.00 (0.06–15.8)
Miscarriage	8 (4)	12 (6)	0.66 (0.28–1.58)
Ectopic pregnancy	0	1 (0.5)	0.33 (0.01–8.06)
Congenital anomalies^1^	2 (1)	8 (4)	0.25 (0.05–1.15)

Data are n (%).

1 After intracervical insemination (ICI): one case of sickle cell anaemia and albinism and one case of trisomy 21.

After IUI: one case of albinism, one case of hypospadias, one case of polydactyly, one case of pelvic ureteric stenosis, one case of anorectal malformation, one case of Turner’s syndrome, one case of West syndrome and one case of Tetralogy of Fallot.

Cycle data are summarized in Table III. In both treatments there were live births until the fifth cycle, to drop strongly in the 6th cycle.

**Table III:** Overview per cycle with logistic regression analysis per cycle.

	Total number of women	Inseminated (n %)	**Not inseminated** **(n %)**	**Did not continue treatment** **(n %)**	Live birth (%)	**OR** **(95% CI)**
**ICI**						
Cycle 1	202	184 (91)	18 (9)	19 (10)	14(8)	1.0
Cycle 2	171	163 (95)	8 (5)	6 (4)	8 (6)	0.66 (0.27–1.61)
Cycle 3	156	140 (90)	16 (10)	6 (4)	12 (9)	1.12 (0.50–2.50)
Cycle 4	131	116 (88)	15 (12)	1 (1)	9 (8)	0.99 (0.41–2.34)
Cycle 5	112	103 (92)	9 (8)	1 (1)	7 (7)	0.91 (0.35–2.31)
Cycle 6	85	77 (91)	8 (9)	0 (0)	1 (1)	0.16 (0.021–1.25)
**IUI**						
Cycle 1	206	187 (91)	19 (9)	14 (7)	24(13)	1.0
Cycle 2	164	149 (91)	15 (9)	3 (2)	28(19)	1.50 (0.83–2.67)
Cycle 3	126	114 (91)	12 (9)	3 (2)	13(11)	0.82 (0.41–1.66)
Cycle 4	108	104 (96)	4 (4)	2 (2)	5(5)	0.41 (0.16–1.04)
Cycle 5	90	79 (88)	11 (12)	3 (3)	9(11)	0.59 (0.25–1.40)
Cycle 6	72	62 (86)	10 (14)	0 (0)	2(3)	0.20 (0.045–0.85)

ICI, intracervical insemination; OR, odds ratio.

Data are n (%), percentages were calculated per women. Percentages for live birth were calculated per insemination.

Logistic regression with cycle one as reference.

**Figure 2.**
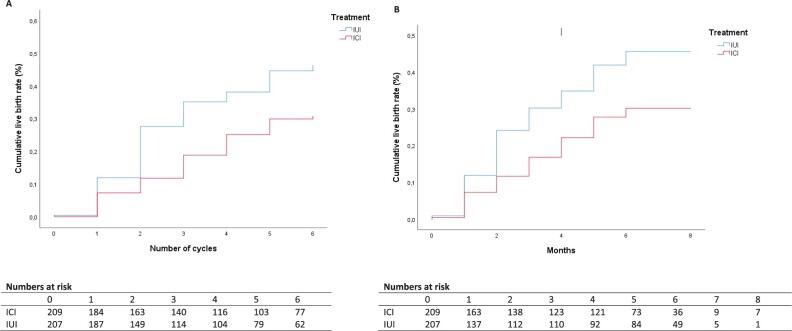
**Time to conception leading to live birth: intention-to-treat analysis.** (**A**) Time to conception leading to live birth (per cycle) corresponding hazard rate for conception leading to live birth 0.58 (95% CI: 0.41 to 0.82). (**B)** Time to conception leading to live birth (months) corresponding hazard rate for conception leading to live birth 0.59 (95% CI: 0.41 to 0.84). ICI, intracervical insemination.

The conception rate leading to live birth within 8 months and over six treatment cycles after ICI and IUI was lower following ICI (log-rank score 10.64, *P* = 0.001 (cycles), log-rank score (months) 9.04, *P* = 0.003) (Fig. 2).

The corresponding HR for conception leading to live birth over six treatment cycles was 0.58 (95% CI: 0.41 to 0.82) for ICI versus IUI.

**Figure 3.**
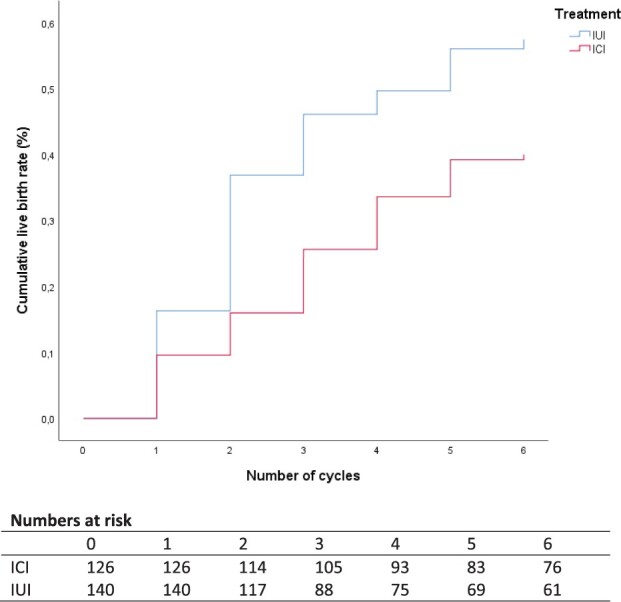
**Time to conception leading to live birth: per-protocol analysis.** Corresponding hazard rate for conception leading to live birth 0.61 (95% CI: 0.43 to 0.87). ICI, intracervical insemination.

Analysis of our pre-specified subgroups showed that our primary outcome did not change according to age or parity (Table IV). The per-protocol population consisted of 126 women allocated to ICI and 140 women allocated to IUI that completed six insemination cycles in their allocated treatment arm or had a live birth. All per-protocol pregnancy outcomes are summarized in Table V. Within the 8 months treatment horizon, 48 women had live births (38%) after ICI and 79 women had live births (56%) after IUI (RR 0.68, 95% CI: 0.52-0.88; RD −18%, 95% CI: −30% to −6%). The conception rate leading to live birth within the 8 months treatment horizon after ICI and IUI over six treatment cycles was lower following ICI (log-rank score 9.66, *P* = 0.002). The corresponding HR for conception leading to live birth was 0.61 (95% CI: 0.43 to 0.87) (Fig. 3).

**Table IV:** Live birth rate in pre-specified subgroups.

	n/N ICI	n/N IUI	RR	CI
Women <35 years old	29/97	46/96	0.62	0.43–0.90
Women ≥35 years old	22/106	33/109	0.69	0.43–1.10
Nulliparous	36/161	69/180	0.58	0.41–0.82
Multiparous	15/48	12/28	0.73	0.40–1.33

ICI, intracervical insemination; RR, relative risk.

**Table V:** Pregnancy outcomes per woman randomized: per-protocol analysis.

	ICI (n = 126)	IUI (n = 140)	Relative risk (95% CI)
Live birth	48 (38)	79 (56)	0.68 (0.52–0.88)
Ongoing pregnancy	50 (40)	80 (57)	0.69 (0.54–0.91)
Clinical pregnancy	54 (43)	84 (60)	0.71 (0.56–0.83)
Multiple pregnancy	1 (0.8)	1 (0.7)	1.00 (0.06–15.8)
Miscarriage	3 (2)	3 (2)	1.11 (0.23–5.40)
Congenital anomalies	1 (1)	7 (5)	0.16 (0.02–1.27)

Data are n (%).

Two women after ICI and one woman after IUI conceived naturally and were excluded in this analysis.

ICI, intracervical insemination.

## Discussion

In this multicentre, non-blinded, randomized controlled non-inferiority trial in women who were eligible for donor sperm treatment, over six cycles ICI was inferior compared to IUI in terms of live birth within the treatment horizon of 8 months.

Our randomized clinical trial has several strengths. First, we included all women with an indication for donor sperm treatment and did not limit our study to any particular family type, thus enhancing the generalizability of our data. Second, we based our power analysis on our large retrospective cohort study and not on outdated results of small RCTs, ensuring robustness of the data (Kop *et al.*, 2015). Third, our per-protocol analysis limited to women that received the allocated treatment and that completed six treatment cycles did not alter our results, suggesting that treatment switches and not completing six treatment cycles did not have an effect on our primary outcome.

One of the limitations in our study is that owing to the nature of the interventions we were not able to blind this study. Also, in our sample size calculation, we did not take into account that women would not start with the assigned intervention or would not complete the six insemination cycles offered. Another concern is that fewer women assigned to ICI were nulliparous compared to women assigned to IUI. Since the chances to become pregnant are lower for nulliparous women, the possible bias caused by these differences in parity would work in the opposite direction of our results (van der Steeg *et al.*, 2008). Finally, there was considerable practice variation in semen processing for IUI and timing of insemination. While this represents daily practice, this enhances the generalizability of our data.

The results of our trial are in line with the only two existing underpowered RCTs, which had clinical pregnancy as primary outcome and were performed almost 30 years ago and therefore perhaps not representative of current practice (Patton *et al.*, 1992; Hurd *et al.*, 1993). In contrast with our retrospective cohort study with an HR of 1.0, our RCT showed an HR of 0.58 after ICI versus IUI. This randomized design probably represents the best estimate of the relative effectiveness.

Our trial shows a strong drop in live birth rate in the sixth cycle after ICI and IUI. Since only a small number of women are present in this analysis, the drop may be due to chance. How many cycles of IUI we should perform before we continue with a second-line treatment could be topic of future research. Additionally, it is unknown what the second-line treatment should be if women do not conceive after IUI in the natural cycle; this could also be a topic for future research.

Thirteen women (3%) decided not to start with the assigned intervention and 137 women (32%) did not complete six treatment cycles within the 8-month’ time horizon. This was mainly because women found their own sperm donor, tried to conceive with a partner or for medical or personal reasons. This underpins that women, even though they had decided to start donor sperm treatment, can make other decisions later on how they fulfil their wish for a child or abstain as they go along. Life is not predictable.

Why ICI is inferior to IUI in these women who—by definition—have no known fertility problems remains a moot point. IUI brings the sperm closer to the oocyte than ICI and this might compensate for decreased sperm motility after freezing and thawing (Keel *et al.*, 1987; Sunde *et al.*, 1988).

Next to effectiveness, treatment costs play an increasingly large role in clinical decision-making (Reinhardt *et al.*, 2004). ICI is cheaper, while processing of donor sperm is not needed and for IUI processing of donor sperm is needed. In view of the large differences in conception leading to live birth between ICI and IUI, it is unlikely that the additional costs for IUI will impact decision-making.

In summary, our study shows that ICI is inferior to IUI in the natural cycle in women undergoing inseminations with cryopreserved donor sperm, in terms of live birth rate. Therefore, IUI in the natural cycle should be the preferred first-line treatment in inseminations with cryopreserved donor sperm.

## Data availability

The data underlying this article cannot be shared publicly due to the privacy of individuals that participated in the study. The data will be shared on reasonable request to the corresponding author.
